# Tolerance of free-living larval stage of a parasite from coastal mining areas in northern Humboldt Current to copper pollution at low and high temperatures

**DOI:** 10.1371/journal.pone.0310473

**Published:** 2024-11-05

**Authors:** Natalia V. Leiva, Diana Montenegro, Rodrigo Orrego, Rodrigo Vidal, M. Teresa González

**Affiliations:** 1 Programa Doctorado en Ciencias Aplicadas Mención Sistemas Acuáticos, Universidad de Antofagasta, Antofagasta, Chile; 2 Instituto de Ciencias Naturales “Alexander von Humboldt”, Facultad de Ciencias del Mar y Recursos Biológicos, Universidad de Antofagasta, Antofagasta, Chile; 3 Centro de Investigación de Estudios Avanzados del Maule (CIEAM), Universidad Católica del Maule, Talca, Chile; 4 Laboratory of Genomics, Molecular Ecology and Evolutionary Studies, Department of Biology, Universidad de Santiago de Chile, Santiago, Chile; Universidade Federal de Minas Gerais, BRAZIL

## Abstract

Metal pollution is a worldwide problem and one of the greatest threats to ecosystem integrity due to its toxicity, persistence, and bioaccumulation in biological systems. Anthropogenic pollution impacts marine organisms and host-parasite dynamics, with the northern Chilean coast experiencing elevated copper levels in marine waters and sediments due to mining activities. In this study, we assessed the effects of exposure to copper concentrations at low and high-water temperatures on the survival and longevity of the marine parasite *Himasthla* sp. cercariae (Trematoda: Digenea) using the snail *Echinolittorina peruviana* as its first intermediate host. Snails were collected from intertidal rocky pools in northern Chile (23°S). To assess parasite survival and longevity, cercariae were collected from a pool of infected snails, and their mortality was recorded every 6 hours until all cercariae were dead. In a preliminary experiment conducted at 19°C, cercariae were exposed to different copper concentrations (0.2, 1.5, 3.0, and 6.0 mg/L) for 78 hours. Cercariae showed tolerance to copper. However, at the higher copper concentration (6 mg/L), survival was negatively impacted (50%) at 54 hours. In contrast, at the lower concentration (0.2 mg/L) and in the control group, cercariae sustained a 73–90% survival rate even after 54 hours. Based on these findings, we conducted subsequent experiments involving two copper treatments (0.2 and 3.0 mg/L) and two temperatures (14 and 22°C). Survival and longevity were significantly higher at lower temperature and copper concentration (14°C and 0.2 mg/L). Conversely, at higher temperature and copper concentration (22°C and 3 mg/L), survival and longevity decreased to only 66 hours. Our results show that *Himasthla* sp. cercariae tolerated most copper concentrations, with vulnerability observed primarily in high water temperatures, indicating an adverse effect on cercariae performance. This study contributes valuable insights into how parasites respond to environmental pollution, in marine ecosystems influenced by anthropogenic activities.

## Introduction

Metal pollution is a worldwide problem and one of the greatest threats to ecosystem integrity due to its toxicity, persistence, and bioaccumulation in biological systems [1, 2]. Metal pollution has been extensively studied in different aquatic ecosystems around the world [3–6]. Discharges from the mining industry, agriculture, and wastewater present high levels of metals such as copper (Cu), cadmium (Cd), zinc (Zn), and lead (Pb) [3]. Cu, like other trace elements such as Zn, iron (Fe), and manganese (Mn), is an essential micronutrient for life due to its role in many important biological processes, including as a cofactor for several key enzymes, electron transfer within biologic al molecules, iron metabolism, free radical scavenging, and various neurological functions [7–9]. At high concentrations, however, these elements can be toxic, causing alterations in motility, growth, and reproduction; disturbed metabolic functions; and eventually, the death of organisms [7, 10, 11]. Additionally, elevated temperatures may increase the availability of heavy metals in aquatic ecosystems because of their solubility [12], and this may act as a driver of toxicity in organisms [13, 14]. Thus, an increase in water temperature combined with metal pollution can generate adverse responses if organisms are not tolerant of or adapted to these conditions.

Host-parasite interactions are an integral part of ecosystems and can influence ecological and evolutionary processes [15, 16]. Parasites play an important role in structuring their host communities [17–19], particularly in closed systems such as intertidal pools, where parasites are recognized as a major factor contributing to the structure of intertidal communities and ecosystem biodiversity [20–22]. Metal pollution and variations in seawater temperature can alter the quality of the environment and influence, directly or indirectly, the responses of parasites (prevalence, intensity, and pathogenicity) and their hosts [23, 24].

Parasites have exhibited alterations due to contamination at all biological organization levels (individual, population, and community) in contaminated environments [25], with trematode parasites being most affected [23, 26–28]. Trematodes are a dominant group of parasites in coastal ecosystems, and their free-living stages (eggs, miracidia, and cercariae larvae) have been described as highly sensitive to a variety of environmental factors (e.g., temperature, lower aquatic pH, and metal exposure), which can, for example, influence the production and release of cercariae into the environment, reduce transmission success to other hosts, and affect their survival and longevity [22–24]. The second free-swimming larval stage of trematodes (cercariae) is the key infective stage in the life cycle of these parasites, playing an important role in their transmission to subsequent hosts [23]. Therefore, it is vital to assess the impact of anthropogenic factors on cercariae’s fitness. Due to parasites’ important role in many ecological processes and population regulation, studies evaluating the effects of environmental factors on parasite larval stages have been increasing [24, 29, 30], but only a few studies have conducted laboratory experiments giving evidence about the effects of metal toxicity on some components of the fitness of larval trematodes especially from freshwater environments, such as cercariae survival [31–33], longevity [34–36], emergence and transmission [37, 38], and activity and orientation behavior [39, 40]. However, the mechanism of Cu toxicity and accumulation in organisms is different in freshwater and seawater due to differences in pH, hardness, alkalinity, temperature, and the presence of other metals. Copper toxicity in algae, invertebrates, and fish generally increases as salinity decreases, with higher accumulation and toxicity in freshwater [41–43]. Despite this differential response, to our knowledge, no previous experimental studies have evaluated the effects of temperature and metal pollution on larval trematode performance in marine systems.

The northern Chilean coast is influenced by the Humboldt Current System (HCS), and it is characterized by its high mining activity and the loading of polymetallic minerals, mainly Cu, Zn, and Pb, and the loading of Cu anodes and cathodes [44, 45]. Scientific studies undertaken in San Jorge Bay, Antofagasta (23°20’S), have recorded high concentrations of Cu in marine waters and sediments, suggesting a relationship between these metals’ presence and the area’s industrial activities [44–47]. The high Cu concentrations recorded in this bay’s water column are comparable to some of the highest levels recorded worldwide, such as in Bangladesh [48], Zhelin Bay in China [5], the coast of Attica in Greece [4], and Chañaral in Chile [44]. Historical records show high levels of Cu in different coastal environments in northern Chile ranging from 0.67 to 2.03 μg/L (0.002 mg/L) [44, 49]. Furthermore, recently, Cu concentrations in water reaching two orders of magnitude higher (200 μg/L = 0.2 mg/L) have been detected in San Jorge Bay [50].

Additionally, in the northern bays of the HCS, it has been reported that seawater temperature in intertidal pools ranges from 15–25°C in summer and 14–18°C in winter, with an annual mean of 18°C [51, 52]. Higher temperatures and elevated metal concentrations may increase stress in organisms, making them more susceptible to or tolerant of such environmental stresses [23, 24, 53]. Tolerance is defined as organisms’ ability to cope with stress, whether due to natural or anthropogenic factors [54]. These capacities are influenced by the environmental conditions that marine organisms face throughout their life cycles, thus retaining the ability to adapt to a stressor when it becomes permanently present [55]. The free-living stages of trematodes are continually exposed to a highly variable environment in the intertidal zone, which carries risks of desiccation and fluctuations in temperature, pH, and oxygen conditions [23, 56]. Therefore, in the face of such conditions, it is expected that the cercariae of *Himasthla* sp., are tolerant to high metal concentrations. The *Himasthla* sp. (Trematoda: Digenea) parasitizes the intertidal snail *Echinolittorina peruviana* as a first intermediate host and constitutes a good biological model due to its high prevalence (10–40%) [24].

Knowing more about parasite responses to anthropogenic and natural environmental fluctuations can help us to better understand their responses to future oceanic conditions such as climate change by predicting parasites’ possible transmission dynamics and their susceptibility to contaminants. Thus, in this study, we experimentally evaluated the effects of single exposure concentrations of Cu and their effects at low and high water temperatures (~14 and 22°C) on the survival and longevity of *Himasthla* sp. cercariae.

## Materials and methods

Stock solutions (1000 mg/L) were prepared from analytical-grade Titrisol® standard copper chloride (CuCl_2_) (Merck Millipore, Darmstadt, Germany) in filtered seawater (10-5-2-μm filters + UV). Copper solutions were diluted to the desired concentrations and added into each corresponding multi-well plate or aquarium.

All plastic and glassware used in this study were washed in 10% nitric acid and hydrochloric acid (HCl) for 24 h and then rinsed with distilled water before use. The was done to ensure that plastics were free from any metal contamination that could alter our results [57].

Seawater parameters such as temperature, salinity, pH, and Cu concentrations were measured in each aquarium at the beginning and end of each copper concentration experiment (Table 1). Seawater pH, temperature and salinity were measured using a HI98194 portable Multi-parameter Water Quality Meter (Hanna Instruments, USA).

**Table 1:** Descriptive summary of the survival and longevity experiments performed in the present study, and mean values ± standard deviation (±S.E.) of the seawater used to characterize the physico-chemical parameters.

	Experiment 1				
Nominal Cu-concentration (mg/L)	Control	0.2	1.5	3	6
N cercariae/replicate	30	30	30	30	30
Replica per treatment	5	5	5	5	5
Exp. Vol. (ml)	10	10	10	10	10
Seawater temperature (°C)	18.28 ± 1.16	18.85 ± 1.16	19.29 ± 0.80	19.02 ± 1.03	19.02 ± 0.53
Measured Cu-concentration (mg/L)	0.09 ± 0.00	0.26 ± 0.02	1.53 ± 0.43	3.13 ± 0.52	6.03 ± 0.45
Salinity (PSU)	35.89 ± 0.44	35.56 ± 0.29	35.44 ± 0.16	35.25 ± 0.48	34.26 ± 0.19
pH	7.95 ± 0.06	7.80 ± 0.04	7.86 ± 0.05	7.73 ± 0.02	7.29 ± 0.09
Longevity Time	T0-T78 (78h)	T0-T78 (78h)	T0-T78 (78h)	T0-T78 (78h)	T0-T66 (66h)
	Experiment 2				
Nominal Cu-concentration (mg/L) and temperature	0.2 /14°C	3 /14°C	0.2 /22°C	3 /22°C	
N cercariae/replicate	40	40	40	40	
Replica per treatment	3	3	3	3	
Exp. Vol. (ml)	10	10	10	10	
Measured temperature (°C)	13.98 ± 0.24	14.63 ± 0.11	22.31 ± 0.74	22.35 ± 0.61	
Salinity (PSU)	32.31 ± 0.14	32.46 ± 0.15	31.85 ± 0.38	31.92 ± 0.71	
pH	7.87 ± 0.07	7.69 ± 0.05	7.78 ± 0.02	7.60 ± 0.05	
Longevity Time (hours)	T0-T198 (198h)	T0-T150 (150h)	T0-T90 (90h)	T0-T66 (66h)	

Nominal Cu-concentrations (mg/L) = Theoretical copper concentrations, N cercariae/replicate = number of cercariae per replicate, Exp. Vol. = volume of seawater used, Measured temperature (°C) = average temperature measured at the beginning and end of each experiment, Measured Cu-concentration = average copper concentration measured at the beginning and end of each experiment, Longevity time = exposure time (hours) of the cercariae to each copper concentration until reaching 100% mortality. Seawater pH, temperature and salinity were measured at the beginning and end of each experiment using a HI98194 portable multi-parameter water quality meter (Hanna Instruments, USA). Copper content in the samples of seawater for all Cu-concentrations and control, were analyzed before toxicity tests for experiment 1 and 2 in the chemistry laboratory ALS Life Sciences Chile (Antofagasta, Chile) (ISO 9001 Certified).

Copper content in the samples of seawater for all Cu-concentrations and control, were analyzed before and after the toxicity tests for experiments in the chemistry laboratory ALS Life Sciences Chile (Antofagasta, Chile) (ISO 9001 Certified) by flame atomic absorption spectrometry (FAAS). Standards methods for the examination of water were used (supplied by High-Purity Standards, USA), and 0.005 mg/L for Cu detection limits were measured.

### Experiment 1

A total of 300 snails were collected from the upper rocky intertidal zone from a site in Coloso on San Jorge Bay (23°45’S, 70°28’W) in July 2024. No permits were required to access the study site for snail collection as it is an area open to the public. Additionally, the collected snails are abundant in the area, and no permit is required for their extraction. Copper values in marine sediments and water in the sampling area averaged 79.8 mg/kg and 0.03 mg/L, respectively—values that are higher than in other areas along the Chilean coast [50, 58, 59]. Snails identified with the parasite *Himasthla* sp. were acclimatized at room temperature (19–20°C) in sterilized seawater for two days before the experiments. The acclimatization period is necessary in order to avoid host stress resulting from field handling, which could alter the subsequent release of cercariae.

**Fig 1:**
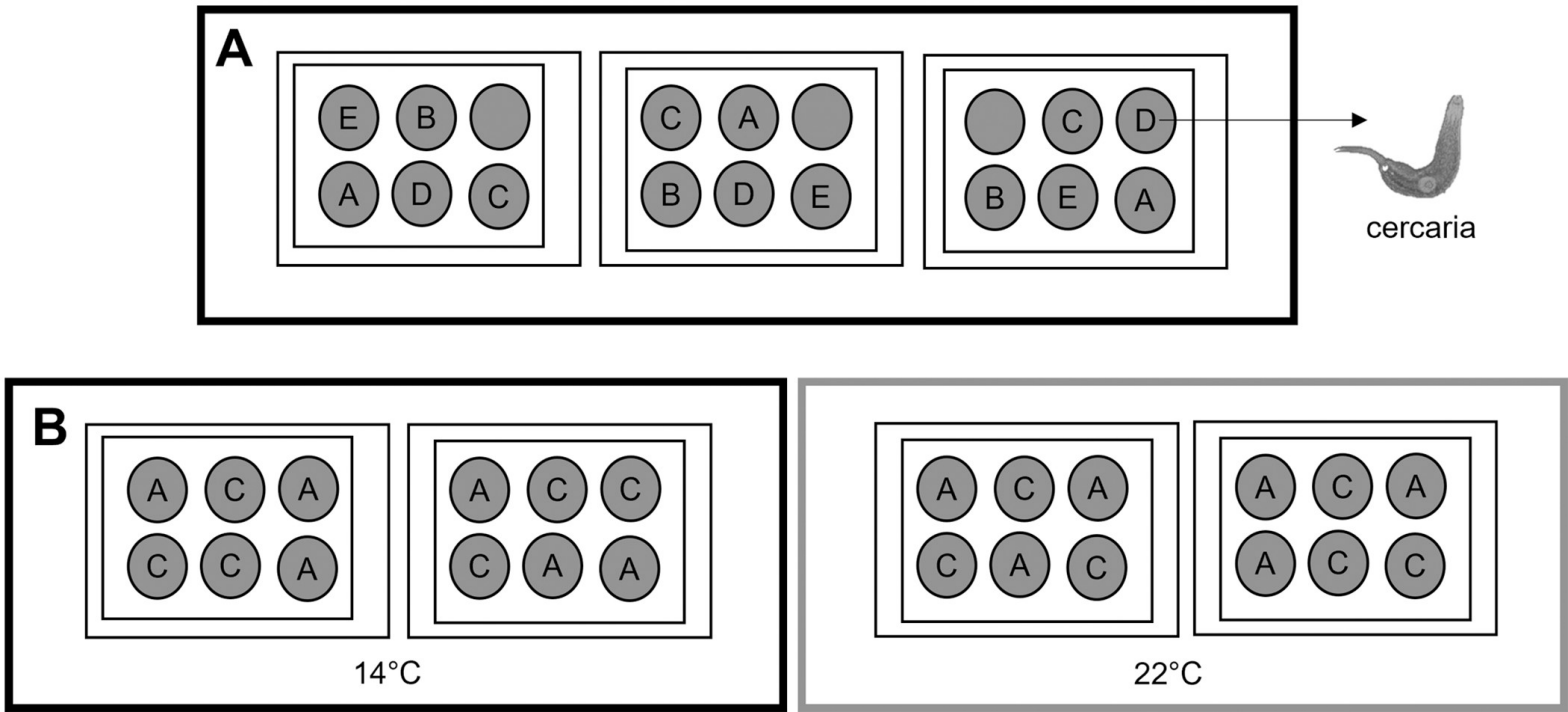
Experimental design for (A) exposure of cercariae to copper, and (B) mixed exposure to copper and temperature (14° and 22°C). A: 0.2 mg/L, B: 1.5 mg/L, C: 3 mg/L, D: 6 mg/L, and E: Control.

To determine the effect of metal concentrations on cercariae’s survival, a preliminary study was carried out using different Cu concentrations and their respective controls: A = 0.2 mg/L, B = 1.5 mg/L, C = 3 mg/L, and D = 6 mg/L (Table 1 and Fig 1). For this, ~750 cercariae of *Himasthla* sp. (age: 2 h) were taken from a pool of infected snails and distributed among 15 multi-well plates (6 wells, 30 cercariae per well). For each treatment, five replicates were used (Fig 1A and Table 1). Cercarial mortalities were reviewed under a stereomicroscope at intervals of 6 h until all cercariae were found to be dead. The 6-hours observation frequency was chosen based on several previous experiments, which began by monitoring cercariae survival every 30 min. To determine cercariae longevity, the mean time between the first observation (time zero = T0) and the last recorded observation (final time = TF) was monitored until 100% mortality was reached (Table 1). Stimulation with a fine needle was used to confirm cercarial death, following Anderson and Whitfield (1975) [60].

### Experiment 2

A total of 300 snails were collected from the study site in March-May 2023. New individuals were collected each time a new experiment was to be conducted. Snails identified with the parasite *Himasthla* sp. were acclimatized at room temperature (18–19°C) in sterilized seawater for two days before the experiments, following Leiva et al. (2017) [24]. The acclimatization period is necessary in order to avoid host stress resulting from field handling, which could alter the subsequent release of cercariae.

In these experiments, Cu-concentration of 6 mg/L was discarded because of the previously established cercarial mortality. Therefore, cercariae were exposed to two Cu-concentrations—A = 0.2 mg/L (natural environmental concentration), and B = 3 mg/L, sub-lethal concentration used in pilot experiments—and two temperature levels: 14°C and 22°C. Temperatures were chosen considering the natural variability existing in intertidal environments of the northern Chilean coast, where temperatures vary between 14.7°C and 25°C [51, 52, 61] (Fig 1B and Table 1). Two ESs were performed (ES1 and ES2). Effects of temperature alone on cercariae survival have been studied previously by Leiva et al. (2019) [24]. These authors determined that cercariae *Himasthla* sp. presents a tradeoff between temperature and survival/longevity, demonstrating higher survival at temperatures (14°C and 18°C) lower than 25°C.

The same criterion of cercarial death used in the first experiment according to Anderson and Whitfield (1975) [60] was established, stimulation with a fine needle. For each ES, ~960 cercariae of *Himasthla* sp. (average age: 2 h) were taken from a pool of infected snails and distributed among 12 multi-well plates (6 wells, 40 cercariae per well). For each treatment, three replicates were used. Cercarial mortalities were inspected with a stereomicroscope at intervals of 6 h until all cercariae were found dead. To determine the cercariae’s longevity, the mean time between the first observation (time zero = T0) and the last recorded observation (final time = TF) was monitored until 100% mortality was reached (Table 1).

### Statistical analysis

For experiment 1, the effects of different Cu-concentrations on cercariae survival were analyzed using generalized linear models (GLMs), with a binomial distribution for the response variable (= cercariae survival/longevity) and logit-link function [62]. In this experiment, exposure time (fixed factor) included from T0 (0h) up to T78 (78 h) nested in treatment (Cu-concentrations) with five replicates per treatment. All replicates were included in the error associated with the statistical model.

Statistical model: Survival = μ+ Cu-concentrations + Time (Cu-concentrations) + e

For experiment 2, the effects of temperature and Cu-concentrations on the survival and longevity of cercariae were analyzed using GLMs, with time and Cu-treatments nested in temperature, using a binomial distribution for the response variable (= cercariae survival/longevity) and logit-link function [58]. In this experiment, the exposure time included from T0 (0h) up to T198 (198 h) with three replicates for treatment. All replicates were included in the error associated to statistical model as replicate’s variability had a negligible effect.

Statistical model: Survival = μ + Temperature + Cu-concentrations (Temperature) + Time (Temperature) + e

The selection of the best models was based on the Akaike criteria. An a-posteriori Dunn test was used to detect significant differences between Cu-concentrations [63].

Additionally, survival analyses (Kaplan-Meier) were performed for each treatment (temperature and Cu-concentrations) to determine the survival/mortality probabilities along the exposure time [64]. All analyses were performed in RStudio version 4.2.3, with a significance level of *p* < 0.05 for all statistical analyses.

## Results

### Effects of metal concentrations on cercariae longevity and survival (Experiment 1)

**Fig 2:**
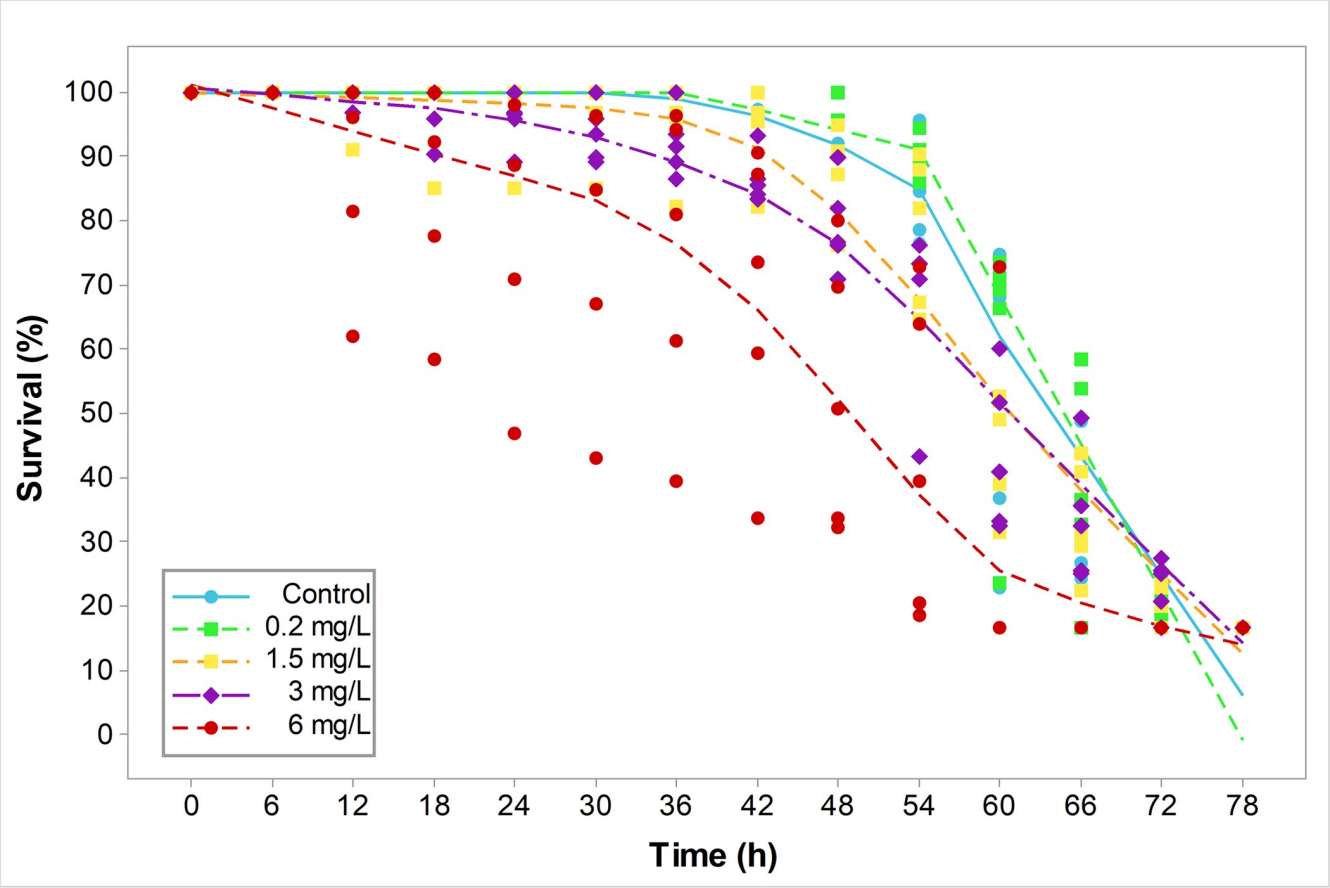
Cercarial survival (%) in response to copper concentrations. Control (blue circle and blue line), and copper concentrations were 0.2 mg/L (green square and dotted green line), 1.5 mg/L (yellow square and orange dotted line), 3 mg/L (purple diamond and purple dotted line) and 6 mg/L (red circle and red dotted line). Each dotted line constitutes a "smoothing line" that is fitted to the data and helps to reveal the potential relationship between variables and predict trends [75].

Cercariae survival varied significantly between Cu-concentrations and exposure time compared with the control group (Table 2 and Fig 2), with the exception of the treatment with the lowest copper concentration (0.2 mg/L). No differences in survival were observed between the 1.5 and 3 mg/L treatments (Dunn test, *p* < 0.246; Table 3). At a higher Cu-concentration (6 mg/L), the first cercarial death was recorded at 12 h of exposure, with survival of nearly 50% (CI: 0.497–0.558, Table 4) at 54 h, and 100% mortality at 66 h (Table 4 and Fig 2). At 1.5 mg/L and 3 mg/L of Cu, the first mortalities were recorded at 12 h, with survival of nearly 50% at 66 h for 1.5 mg/L (CI: 0.523–0.596, Table 4) and 3 mg/L (CI: 0.464–0.551, Table 4), and 100% mortality at 78 h (Table 4 and Fig 2). At the lowest Cu-concentration (0.2 mg/L) and in the control group, the first mortalities were recorded at 48 h and 42 h, respectively, with survival of nearly 50% (0.2 mg/L: CI: 0.637–0.707, control: CI: 0.534–0.612) at 66 h, and 100% mortality at 78 h (Table 4 and Fig 2).

**Table 2:** Generalized Linear Models (GLMs) summary of the effects of copper concentrations, exposure time, and temperature on cercarial survival and longevity of *Himasthla* sp.

		Factor	Chi	d.f.	Resid df	Resid Dev	*P* Chi
EXP.1	SE1	Cu	75.6	4	11534	13392	**< 0.001**
		Time (Cu)	8227.8	63	11471	5194	**< 0.001**
		NULL			11538	13467	
EXP.2	SE1	Te	13.1	1	8651	10306.8	**< 0.001**
		Cu (Te)	305.9	2	8649	10000.9	**< 0.001**
		Time (Te)	6603.6	33	8616	3397.3	**< 0.001**
		NULL			8652	10320	** **
	SE2	Te	59.6	1	8867.3	11371.5	**< 0.001**
		Cu (Te)	229.9	2	8637.4	10881.8	**< 0.001**
		Time (Te)	4576	34	4061.4	2519.5	**< 0.001**
		NULL			8926.9	11775.8	

The effects of copper (Cu) concentration, exposure time (hours), and temperature (Te,°C) were evaluated, with time (Time (Te)) and treatments (Cu (Te)) nested in temperature using a binomial distribution for the response variable (= cercariae survival/longevity) and logit-link function [62]. The analyses were conducted separately for each experiment (EXP) and for each experimental series (ES). The GLMs used in this study were performed following Venables and Ripley (2002) [62]. Significant effects are highlighted in bold (*p* <0.05).

**Table 3:** Dunn test of the effects of copper concentrations (0.2, 1.5, 3, and 6 mg/L) on cercarial survival and longevity of *Himasthla* sp. in experiment 1.

	Control	0.2 mg/L	1.5 mg/L	3 mg/L	6 mg/L
Control					
0.2 mg/L	0.061				
1.5 mg/L	**0.033**	**< 0.001**			
3 mg/L	**0.002**	**< 0.001**	0.246		
6 mg/L	**< 0.001**	**< 0.001**	**< 0.001**	**< 0.001**	

Significant effects are highlighted in bold (*p* <0.05).

**Table 4:** Survival analyses (Kaplan-Meier) of cercariae *Himasthla* sp. showing survival proportion over time when exposed to different acute copper concentrations (0.2, 1.5, 3, and 6 mg/L) at 18.9 ± 0.38°C.

Treatment	Time (h)	Survival	std. Err	Lower 95% CI	Upper 95% CI
Control	T42	0.999	0.001	0.097	1.000
	T48	0.991	0.003	0.985	0.997
	T54	0.957	0.007	0.943	0.970
	T60	0.803	0.015	0.774	0.833
	**T66**	**0.572**	**0.019**	**0.534**	**0.612**
	T72	0.293	0.018	0.258	0.333
	T78	0			
0.2 (mg/L)	T48	0.998	0.001	0.996	1.000
	T54	0.974	0.005	0.965	0.984
	T60	0.867	0.012	0.844	0.890
	**T66**	**0.671**	**0.017**	**0.637**	**0.707**
	T72	0.344	0.019	0.308	0.385
	T78	0			
1.5 (mg/L)	T12	0.999	0.001	0.997	1.000
	T18	0.996	0.001	0.993	0.999
	T24	0.993	0.002	0.989	0.997
	T30	0.989	0.002	0.984	0.994
	T36	0.982	0.003	0.976	0.989
	T42	0.975	0.004	0.967	0.983
	T48	0.953	0.006	0.942	0.965
	T54	0.908	0.009	0.890	0.926
	T60	0.762	0.001	0.733	0.792
	**T66**	**0.559**	**0.001**	**0.523**	**0.596**
	T72	0.294	0.002	0.261	0.332
	T78	0			
3 (mg/L)	T12	0.999	0.001	0.998	1.000
	T18	0.996	0.001	0.993	1.000
	T24	0.991	0.002	0.985	0.996
	T30	0.982	0.004	0.974	0.990
	T36	0.969	0.005	0.959	0.980
	T42	0.947	0.007	0.932	0.961
	T48	0.906	0.010	0.886	0.927
	T54	0.828	0.014	0.800	0.857
	T60	0.687	0.019	0.650	0.726
	**T66**	**0.506**	**0.002**	**0.464**	**0.551**
	T72	0.272	0.002	0.234	0.317
	T78	0			
6 (mg/L)	T12	0.984	0.002	0.979	0.990
	T18	0.965	0.004	0.956	0.973
	T24	0.936	0.005	0.924	0.947
	T30	0.899	0.007	0.884	0.913
	T36	0.852	0.009	0.834	0.870
	T42	0.788	0.001	0.766	0.810
	T48	0.679	0.013	0.653	0.706
	**T54**	**0.527**	**0.015**	**0.497**	**0.558**
	T60	0.304	0.015	0.275	0.337
	T66	0			

The times (T) shown correspond to the number of hours until the cercariae begin to die and until 100% mortality is reached. Time (hours) to >50% probability of cercariae survival is highlighted in bold.

### Effects of temperature and Cu on cercariae longevity and survival (Experiment 2)

**Fig 3:**
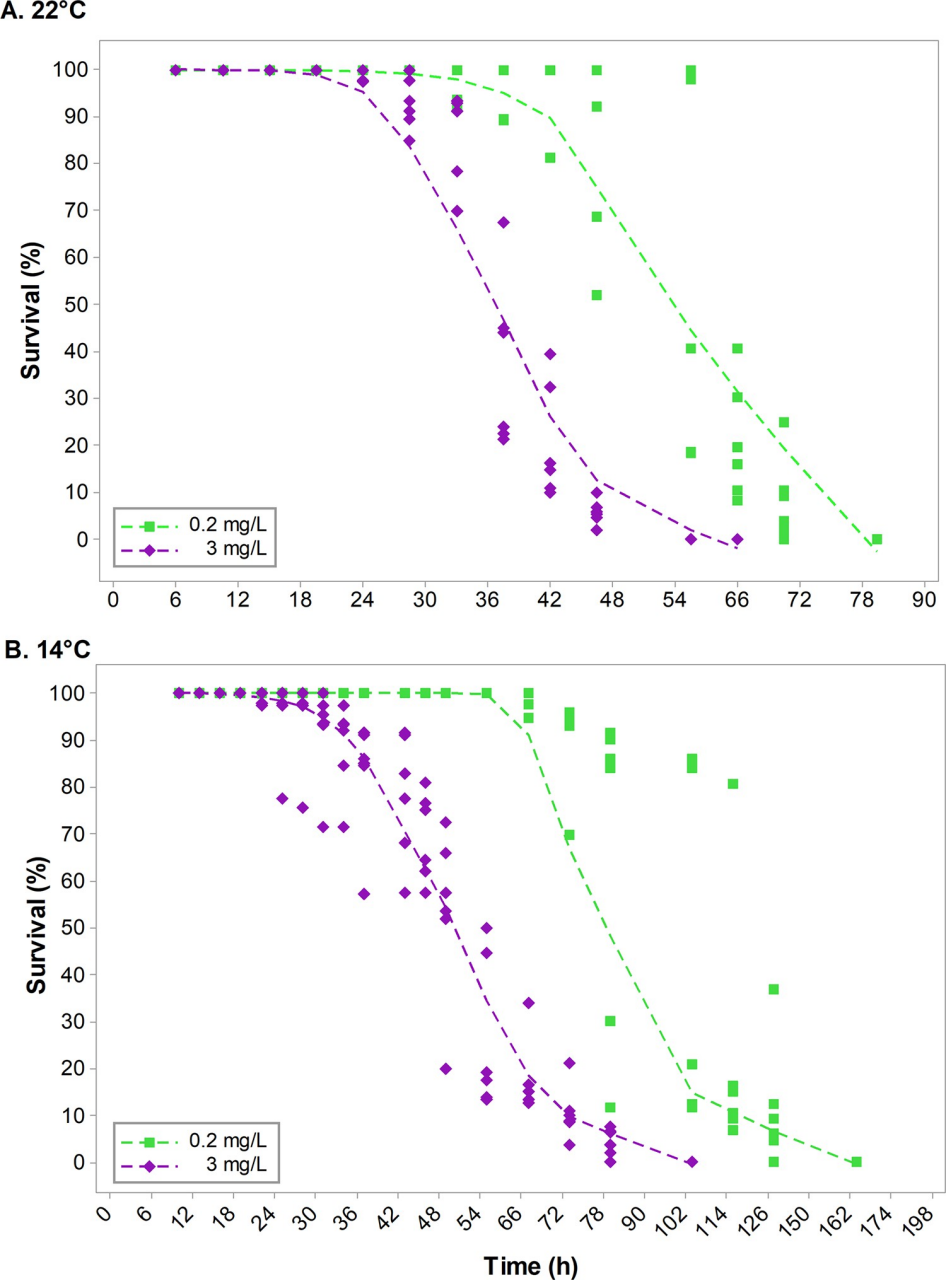
Cercarial survival (%) in response to copper concentrations and temperature (A: 22°C, and B: 14°C). Metal treatments were Control (0.2 mg/L, green square and green dotted line) and 3 mg/L (purple diamond and purple dotted line). Each dotted line constitutes a "smoothing line" that is fitted to the data and helps to reveal the potential relationship between variables and predict trends [75].

Significant effects of temperature, metal concentration, and exposure time were observed on cercariae survival and longevity for *Himasthla* sp. in both ES1 and ES2 (Table 2 and Fig 3). The slight natural variability of the replicates did not affect the overall response in cercariae survival (Fig 3). At a higher temperature (22°C) and Cu concentration (3 mg/L), the first cercarial death was recorded at 24 h of exposure, with survival of nearly 50% (CI: 0.544–0.610, Table 5) at 48 h, and 100% mortality at 66 h (Table 5 and Fig 3A). At 22°C and a lower Cu concentration (0.2 mg/L), the first mortalities were recorded at 36 h, with only 50% of cercariae (CI: 0.597–0.660, Table 5) surviving after 72 h of exposure and reaching 100% mortality at 90 h (Table 5 and Fig 3A). On the other hand, a low temperature (14°C) and 0.2 mg/L Cu caused the first mortalities only at 102 h, reaching 100% mortality at 198 h, while at the 3 mg/L Cu concentration, 50% (CI: 0.531–0.576, Table 5) of cercariae survived up to 102 h, reaching 100% mortality at 162 h (Table 5 and Fig 3B). Consequently, survival and longevity were notably diminished at increased temperature (22°C) and high Cu-concentration (3 mg/L).

**Table 5:** Survival analyses (Kaplan-Meier) of cercariae *Himasthla* sp. exposed to Cu concentrations (0.2 and 3 mg/L) and water temperatures (14 and 22°C).

Temperature	Treatment	Time (h)	Survival	std. Err	Lower 95% CI	Upper 95% CI
14°C	0.2 mg/L	T102	0.997	0.001	0.994	0.999
		T114	0.982	0.003	0.975	0.988
		T126	0.917	0.007	0.902	0.931
		T150	0.804	0.011	0.783	0.826
		**T162**	**0.605**	**0.014**	**0.577**	**0.635**
		T174	0.343	0.015	0.315	0.375
		T198	0	0.003	0.002	0.017
	3 mg/L	T24	0.999	0.000	0.998	1
		T30	0.995	0.001	0.993	0.998
		T36	0.991	0.002	0.988	0.994
		T42	0.984	0.002	0.980	0.988
		T48	0.973	0.002	0.968	0.979
		T54	0.955	0.004	0.948	0.962
		T66	0.929	0.005	0.912	0.939
		T72	0.890	0.006	0.877	0.902
		T78	0.824	0.008	0.801	0.840
		T90	0.711	0.010	0.692	0.731
		**T102**	**0.553**	**0.012**	**0.5312**	**0.576**
		T114	0.397	0.012	0.375	0.421
		T126	0.219	0.010	0.200	0.240
		T150	0.100	0.007	0.082	0.111
		T162	0			
22°C	0.2 mg/L	T36	0.997	0.001	0.995	0.999
		T42	0.992	0.002	0.988	0.996
		T48	0.983	0.003	0.976	0.989
		T54	0.955	0.005	0.944	0.966
		T66	0.871	0.010	0.852	0.890
		T72	0.628	0.016	0.597	0.660
		**T78**	**0.309**	**0.016**	**0.279**	**0.342**
		T90	0			
	3 mg/L	T24	0.999	0.0001	0.998	1
		T30	0.983	0.004	0.976	0.990
		T36	0.956	0.005	0.945	0.968
		T42	0.799	0.013	0.774	0.825
		**T48**	**0.5976**	**0.016**	**0.544**	**0.610**
		T54	0.305	0.016	0.275	0.339
		T66	0			

The times (T) shown correspond to the number of hours until the cercariae begin to die and until 100% mortality is reached. Time (hours) to 50% probability of cercariae survival are highlighted in bold.

## Discussion

In marine environments, host-parasite relationships are affected by multiple environmental factors such as temperature, salinity, ultraviolet radiation, and metal pollution [23, 65]. Furthermore, the toxicity and bioavailability of copper can vary between fresh and seawater due to differences in pH, hardness, alkalinity, temperature, and the presence of other metals [41, 43, 66]. Therefore, organism survival may vary between aquatic environments. To our knowledge, no previously published studies have evaluated the combined effect of metal exposure and temperature on cercarial survival and longevity in marine systems. Leiva et al. (2019) [24] evaluated the individual effect of temperature on the survival of *Himasthla* sp. The authors demonstrated that only the highest temperature (25°C) negatively affected the survival of cercariae, while a temperature of 18°C (the temperature used in the first experiment of the present study) did not have a significant effect. This result is consistent with the results of the first experiment. However, the combined effect of the temperature (18°C) and copper concentrations (1.5, 3, and 6 mg/L) increased the mortality of *Himasthla* cercariae by 50% at 60 hours of Cu-exposure. Our results showed that free-living larval stages of digenean *Himasthla* sp. tolerated high Cu concentrations (two orders of magnitude higher than Cu concentrations historically recorded as background in waters of the northern Chilean coast), with the cercariae’s vulnerability to this metal exposure increasing only as temperature increases as well, revealing a negative effect of temperature and Cu exposure on cercariae’s performance in marine systems.

Environmental pollution can negatively influence the survival and longevity of distinct larval parasite stages by reducing their success of transmission to other hosts and altering their life cycles [22, 24, 28]. Previous studies have reported a general trend of decreased survival and longevity in cercariae from freshwater environments when toxicant concentration increases. In this regard, Cross et al. (2001) [34] evaluated the effects of acute toxicity of metals (Cu, Zn, Fe, and Mn) on the traits of cercariae infecting the freshwater snail *Cryptocotyle lingua* and found a negative effect on their longevity, measured as the midpoint between the first observation (T0) and the final observation (TF = dead), and swimming speed (measured in mm/sec traveled in a swimming chamber). Similarly, Evans (1982) [67] demonstrated that high Cu and Zn concentrations led to a reduction in cercarial survival and infectivity of the freshwater trematode *Echinoparyphium recurvatum*, recording 50% survival after 20 h exposure and 30 h of maximum lifespan. Like previous studies, Reddy et al. (2004) [68] demonstrated that high concentrations of CuSO_4_ resulted in a significant decrease in the survival of cercariae of the freshwater species *Echinostoma caproni* and *Echinostoma trivolvis*, reaching 100% mortality within the first two hours of exposure. Additionally, Ibrahim et al. (2022) [69] examined the toxic effect of CuO on the survival of larval stages (miracidium and cercaria) of the freshwater trematode *Schistosoma mansoni*, demonstrating that prolonged exposure and higher concentrations of this metal resulted in high and rapid mortality within 10 minutes of exposure. On the other hand, Morley et al. (2001) [70] experimentally assessed the effects of the metals Zn and Cd, as well as water temperatures (10, 20, and 25°C), on the cercarial survival of freshwater digenean *Diplostomum spathaceum*. These authors found higher survival rates for both the control group and the metal treatments at lower temperatures, possibly due to reduced cercarial metabolism at lower temperatures, leading to lower utilization of finite glycogen reserves. These results demonstrate that parasites’ responses can vary depending on the type of metal as well as the combined effects of other environmental factors such as temperature, which can increase or decrease the effects of metal toxicity [39, 70].

For a long time, it has been reported that water Cu concentrations on the northern Chilean coast are similar to those of various coastal environments worldwide with similar anthropogenic intervention (0.5 to 2.0 μg/L) [44]. The copper levels currently recorded in the study area exceed by two orders of magnitude those previously reported in the water of San Jorge Bay, with concentrations above 0.2 mg/L (unpublished data), values that would exceed the Chilean national standard for water quality published in the seawater quality guideline, categorized as class 3 (regular quality) [71, 72]. Interestingly, in our study, cercariae of *Himasthla* sp. were highly tolerant of different concentrations of Cu, with their survival only being negatively affected when exposed to the highest treatment (6 mg/L), reaching a 50% mortality rate after 48 h of exposure. Additionally, exposure to high temperature and copper concentration (22°C and 3 mg/L) had a synergistic negative effect on the survival of *Himasthla* sp. (Fig 3). However, this effect was less significant when cercariae were exposed to a high Cu concentration at a lower water temperature, demonstrating that this species’ larval stages can tolerate the high concentrations of metals present in this bay. Moreover, it has been suggested that free-living stages of parasites can withstand environmental variations [23], with some cercariae exhibiting the ability to tolerate environmental conditions similar to those of their hosts. This may apply to trematode species infecting the snail *E*. *peruviana*, which under natural conditions inhabits the rocky intertidal zone, where it is exposed to variations in temperature, desiccation, and pH, and other factors, and is also characteristically tolerant of such conditions [56, 65]. This metal tolerance could be an indication of the parasite species’ adaptive capacities, with an increased cercarial survival potentially benefiting infectivity success with the next host.

## Conclusion

In summary, although at an experimental scale, this study suggests that the cercariae of *Himasthla* sp. tolerate copper concentrations higher than those recorded in the environment and can survive up to 8 days (198 hours) at a concentration of 0.2 mg/L and low temperature (14°C). Additionally, seawater cercariae appear to be less sensitive to similar concentrations of Cu compared to other freshwater cercariae species [62–64], as they can survive for a longer period under prolonged exposure to this metal. The increased survival and longevity of cercariae of this species could potentially have cascading effects on ecological processes and ecosystem function, as this parasite regulates host snail populations through castration [73, 74].

Further research is needed to elucidate the molecular mechanisms (e.g., characterization of metabolic functions and presence of metal resistance genes) by which this parasite tolerates high concentrations of Cu or other metals, using advanced molecular tools such as transcriptomics, metabolomics, and metagenomics. Consequently, our results, along with new approaches, will allow us to generate knowledge on how parasites respond to environmental pollution, allowing us a better understanding and prediction of parasite infection dynamics in marine environments with strong anthropogenic influences.

## Supporting information

S1 File (XLSX)
